# The role of a multidisciplinary clinic for management of patients with IDH mutant gliomas

**DOI:** 10.3389/fonc.2025.1729589

**Published:** 2025-12-17

**Authors:** Lauren Aucoin, Jessica W. Templer, Matthew C. Tate, Karan S. Dixit

**Affiliations:** 1 Department of Neurology, Northwestern University, Feinberg School of Medicine, Chicago, IL, United States; 2 Department of Neurosurgery, Northwestern University, Feinberg School of Medicine, Chicago, IL, United States; 3 Lou and Jean Malnati Brain Tumor Institute, Robert H. Lurie Comprehensive Cancer Center, Feinberg School of Medicine, Northwestern University, Chicago, IL, United States

**Keywords:** IDH mutant glioma, low grade glioma, multidisciplinary care, quality of life, tumoral epilepsy

## Abstract

**Introduction:**

Management of IDH-mutant lower-grade gliomas (IDHm LGGs) is complex, requiring careful balance between tumor control as well as neurocognitive function and quality of life. While multidisciplinary clinics improve outcomes in other cancers, no structured model exists for IDHm LGGs.

**Methods:**

We present a framework for a multidisciplinary clinic a dedicated to patients with IDHm LGGs.

**Discussion:**

IDHm LGGs, including WHO grade 2 astrocytomas and oligodendrogliomas, affect younger adults with median survivals exceeding a decade. Tumor related seizures are a significant source of distress for patients with IDHm LGGs, in addition to burden of disease. Treatment decisions such as surgery, medical therapy, and radiation, along with seizure management are highly individualized and benefit from coordinated, longitudinal care. A dedicated multidisciplinary LGG clinic integrates expertise across neurosurgery, neuro-oncology, epilepsy, and neuropsychology to optimize outcomes and patient experience to create a onco-functional balance.

**Conclusion:**

A multidisciplinary clinic dedicated to IDHm LGGs can provide a collaborative model for comprehensive, patient-centered care for a rare and complex disease which may be replicated at other brain tumor centers.

## 1 Introduction

As our knowledge of disease pathophysiology and management option increases, patient care becomes increasingly intricate and collaborative. Multidisciplinary clinics have been utilized as a method for providing care to patients with complex and rare diseases and have been associated with improved adherence to clinical guidelines, as well as subsequently reduced morbidity and mortality (1, 2). Prior research has evaluated the benefits of multidisciplinary clinics for patients with glioblastoma, however, at this time there are no documented frameworks of multidisciplinary clinics for patients with IDH mutant lower-grade gliomas (3, 4).

Adult-type diffuse gliomas were redefined in 2021 by the World Health Organization (WHO CNS5) as having either the presence or lack of an isocitrate dehydrogenase (IDH) mutation. Lower-grade gliomas (LGG) have a mutation in isocitrate dehydrogenase 1 or 2 (IDHm) and specifically include WHO grade 2 oligodendrogliomas and WHO grade 2 IDHm astrocytomas (5, 6). Between 2018 and 2021 in the United States, 1,675 patients were diagnosed with grade 2 IDHm astrocytomas, and 1,885 patients were diagnosed with grade 2 IDHm oligodendrogliomas. The median age of diagnosis for patients with grade 2 IDHm astrocytomas was 35 years old, and 42 for patients with grade 2 oligodendrogliomas (7). The median survival for patients with grade 2 astrocytomas was 11.6 years, and 17.8 years for patients with grade 2 oligodendrogliomas (6–8). Given the earlier age of diagnosis and longer-term prognosis, the management considerations for patients with IDHm lower-grade gliomas are distinct from those with higher grade gliomas.

The goal of management for patients with LGG is to develop an onco-functional balance of tumor control, seizure control, neurocognition, and functional preservation, as well as further extending survival. Initial treatment is maximal safe resection, which is associated with better outcomes as opposed to subtotal resection or no resection (9–11). Following maximal safe resection, several factors are considered to determine patient risk profile and subsequent further management. These factors include, but not limited to, extent of resection, tumor histology and molecular profile, age, and seizure burden. For patients who are considered lower risk, such as patients with gross total resection extent resection, oligodendroglioma, and age < 40, a “watch and wait” strategy may be appropriate. This approach entails patients undergoing serial neuroimaging with MRIs to monitor tumor growth, with the goal of delaying radiation or chemotherapy until radiologic or clinical progression necessitate these measures (9). However, for patients deemed to be at higher risk, medical management and radiation is used as the combination has been shown to increase both overall survival and progression free survival (9, 12, 13). With the regulatory approval and availability of vorasidenib, a brain permeable mutant IDH 1/2 IDH inhibitor, there is now an additional medical option for patients with WHO grade 2 IDHm glioma who would benefit from therapy but who may not yet need more aggressive chemotherapy and radiation (14). An additional important facet of treatment for patients is seizure management; 70-90% of patients with IDHm LGGs have at least one seizure (15). Given the range of necessary treatments and options, management of IDHm gliomas is inherently multidisciplinary and hence requires collaboration across multiple medical specialties for comprehensive care.

Patients with LGGs face a variety of unique challenges, which complicates treatment and patient care. Even prior to treatment, patients with LGGs experience cognitive impairments across multiple domains, most prominently in attention (16). Prior research has demonstrated that radiation therapy, regardless of fraction dose, is associated with decreased cognitive functioning in multiple domains for patients with LGGs (17). Furthermore, contemporary evaluation of patients with IDHm glioma suggests that early use of radiotherapy may not actually improve patient outcomes (18). In addition, the use of temozolomide chemotherapy can lead to a more aggressive hypermutated phenotype when disease progression occurs (19). Given that patients with LGGs regularly live for over a decade post diagnosis, long term sequelae of therapy must be considered, and hence the decision of when chemotherapy and radiation is appropriate is individualized to each patient and takes into consideration both tumor burden and quality of life concerns. Providers must also consider the ways in which potential repeat resection of patients’ LGGs may secondarily impact the timing of medical therapy and radiation, as repeat resection has been demonstrated to both improve outcomes and preserve patient quality of life (20, 21). Additionally, seizures, which most patients with LGG experience, are consistently associated with decreased quality of life in patients with primary brain tumors (15, 16, 22, 23). Further adding to this challenge, patients with LGGs frequently have refractory seizures which require multiple medications, ongoing titrations, and continuous adjustments (15, 23). Given the constantly evolving understanding about glioma biology, complexities of providing individualized longitudinal care, and challenges to quality of life that LGGs pose, an innovative and collaborative solution is needed to best serve these patients. Here, we detail a model for an LGG clinic, discussing advantages of the model and applicability to other institutions.

## 2 Clinic model

The medical team in the clinic is composed of a neurosurgeon, neuro-oncologist, and epileptologist specializing in tumor associated epilepsy. Other providers and members of the team include a neuropsychologist with expertise in brain tumors, social worker, pharmacist, and a nurse coordinator. The clinic occurs once a month with a census of 5–8 patients in a half day. Patients are seen at differing frequencies depending on their stage of disease and needs. For example, patients with newly diagnosed disease or those earlier in their disease course may be seen more frequently, up to every 4–6 months, while those with stable disease are seen every 8–12 months. Prior to each clinic visit, patients undergo neuroimaging, which the clinicians review for any changes and perform volumetric analysis, which are tracked longitudinally. Currently, volumetrics are manually obtained by the neurosurgeon and/or neuro-oncologist; however, semi-automated volumetrics are being implemented to simplify the process. Depending on patient specific needs, neuropsychological evaluation is obtained serially, every 1 to 2 years, before planned surgery, or more frequently if necessary. Prior to seeing each patient, the team reviews the latest neuroimaging and compares it to both recent and remote studies to assess for rate volumetric growth, and discusses the management plan from surgical, medical, seizure, and neuropsychological perspectives. The patient visit consists of all three physicians, as well as other trainees and staff, to facilitate a true multidisciplinary conversation where the medical team has sufficient time to obtain thorough histories and provide counseling, and for patients and caregivers to have ample time, as well as an open environment, to ask questions about their disease and management strategy. If necessary, the neuropsychologist also meets with the patient separately to review testing results and provide counseling if needed. Finally, patients also have access to a social worker who meets with them separately.

## 3 Benefits

### 3.1 Efficiency

A multidisciplinary clinic fosters increased efficiency of care for patients with LGGs. Patients receive management and counseling from multiple providers concurrently throughout their disease course which ensures that there is a unified and direct plan. Surgery, medical therapy, and seizure control are all managed by different physicians yet treatment changes in one domain are dependent upon coordination with other subspecialities. Although this is possible in a multiple appointment model, this means providers must be constantly in communication about updates, often asynchronously and virtually. As a result, this can lead to delays in treatment and additional unnecessary appointments for which patients must miss work, important in the context that up to 90% of patients with LGGs continue to work (24). In the multidisciplinary clinic, clinicians collaborate in real time to form cohesive treatment plans which balance each domain of care. These benefits are not just theoretical; in other multidisciplinary clinics for patients with cancer, this type of collaboration is associated with decreased delays in time to treatment following diagnosis, and increased patient satisfaction as compared to traditional models (2, 25). Given the longitudinal nature of LGG care, increased efficiency and decreased delays in treatment are even more paramount.

### 3.2 Epilepsy management

An additional unique feature of the multidisciplinary LGG clinic is the presence of an epileptologist who specializes in brain tumor related epilepsy (BTRE), available for every patient with a history of seizures. Seizures are present in most patients with LGGs and can prevent patients from engaging in activities of daily life such as driving, working, or athletic activities, and may be life threatening (22, 26–29). Importantly, while auras (i.e. subjective symptoms) alone may not be deemed ‘significant’ by a provider, the impact of focal seizures and auras may cause significant emotional distress to patients, both due to patient fear over when the next seizure will occur, as well as the seizure acting as a representation and reminder of their glioma (16, 22). Given these impacts, it is unsurprising that seizures are the leading quality of life concern for patients with IDHm gliomas (26). Seizure management for patients with LGGs is nuanced and challenging. Up to 33% of patients do not have seizure control on initial management and ultimately up to 40% of patients with BTRE are determined to have refractory epilepsy, having uncontrolled seizures despite trying 2 or more medications (29–34). Adding to the complexity of seizure management are the concurrent oncologic treatments such as gross total resection, radiotherapy, and chemotherapy all are associated with decreased seizure burden (23, 33, 35). Therefore, antiseizure medication regimens should be re-evaluated on an ongoing basis. Finally, ongoing research suggests that 2-hydroxyglutarate, a product resulting from the IDH mutation, promotes a hyperexcitable environment, and hence novel medicines inhibiting mutant IDH may decrease seizures (28). Given the complexity of navigating BTRE in patients with LGGs, our epileptologist focused specifically on BTRE is a key force driving development of individualized treatment plans that evolve with patients.

### 3.3 Ability to effectively address Quality of Life needs in a population with those needs often unmet

Patients with IDH mutant gliomas face a broad spectrum of unique challenges, often reporting poor quality-of-life (QoL) overall (26). Concerns include financial burdens, challenges with returning to work, and lifestyle restrictions resulting from seizures (24, 27). Other challenges include the impact of disease and treatment on patients’ cognition over the long term (36). Although the magnitude and significance of QoL concerns in this patient population have been characterized, further research is needed to determine how to best identify and address these concerns within the time constrictions of a standard visit model. The multidisciplinary clinic can bridge this gap in knowledge through incorporating multiple specialists focused on addressing quality of life concerns, ensuring concerns are both detected and addressed in a timely manner. One such service is neuropsychology. Our neuropsychologist is trained in brain tumors and awake craniotomies and meet with patients both prior to surgery as well as following surgery and throughout treatment. Prior research has demonstrated neuropsychology’s sensitivity at detecting a range of cognitive impairments in patients with gliomas (37). The clinic also works with a dedicated social worker for patients with brain tumors to address financial concerns and other social determinants of health. A centralized setting with multiple expert practitioners considering quality life concerns in all dimensions of a patient’s care allows for more thorough and timely detection and ability to address concerns, especially important given the lack of research on best practices to standardize detection.

### 3.4 Research

The centralized multidisciplinary clinic model also fosters additional clinical research. A large population of patients with a rare disease concentrated in one clinic demonstrates a full spectrum of presentations, challenges, and treatment responses. Research is ongoing in multiple fields, including quality of life, seizure control, and other best practices, and allows patients to access cutting edge treatments through clinical trials that would not otherwise be available.

### 3.5 Referral site

Finally, the multidisciplinary clinic acts as a tertiary referral site for patients and physicians in the community. Access to multiple physicians at once allows patients to easily obtain management opinions, which are then communicated directly to their external treating team or referring physician in the form of a summary letter containing recommendations from each treating physician as well as volumetric growth pattern of the patient’s tumor.

## 4 Conclusion and adaptation to other institutions

Multidisciplinary clinics in other medical fields have thus far demonstrated benefits pertaining to patient outcomes, patient perspectives on care, and quality of life (1, 2, 4, 25). Patients with brain tumors, including those with LGGs, unfortunately experience acute exacerbations or changes in their disease status which need to be evaluated and managed in a rapid fashion, and hence are uniquely suited to a multidisciplinary program. We continue to consider future directions to improve our model, including the addition of a radiation oncologist to enhance multidisciplinary decision-making and optimize treatment planning for our patients. In addition, we are collecting data to assess the true impact of multidisciplinary care, such as improvements in outcomes, quality of life, patient satisfaction, and healthcare expenditure. We also aim to further characterize the range of unmet needs of our patients with LGGs. This research will help inform best practices, not only in prolonging survival but in supporting and improving patient quality of life.

Here we provide the first description of a multidisciplinary clinic specifically focused on LGGs. Although a dedicated clinic focused specifically on LGGs may not be practical at every institution, elements of our multidisciplinary program can likely be incorporated in other centers with brain tumor programs to better serve this unique patient population and improve their care.

## Data Availability

The original contributions presented in the study are included in the article/supplementary material. Further inquiries can be directed to the corresponding author/s.
